# Supplementary material for: First-principles quantum dynamical theory for the dissociative chemisorption of H2O on rigid Cu(111)
Source: Nat Commun. 2016 Jun 10;7:11953. doi: 10.1038/ncomms11953 (PMC4906410; doi:10.1038/ncomms11953)
Supplement: Supplementary Information — Supplementary Figures 1-7, Supplementary Discussion and Supplementary References. [file ncomms11953-s1.pdf]

I. SUPPLEMENTARY FIGURES

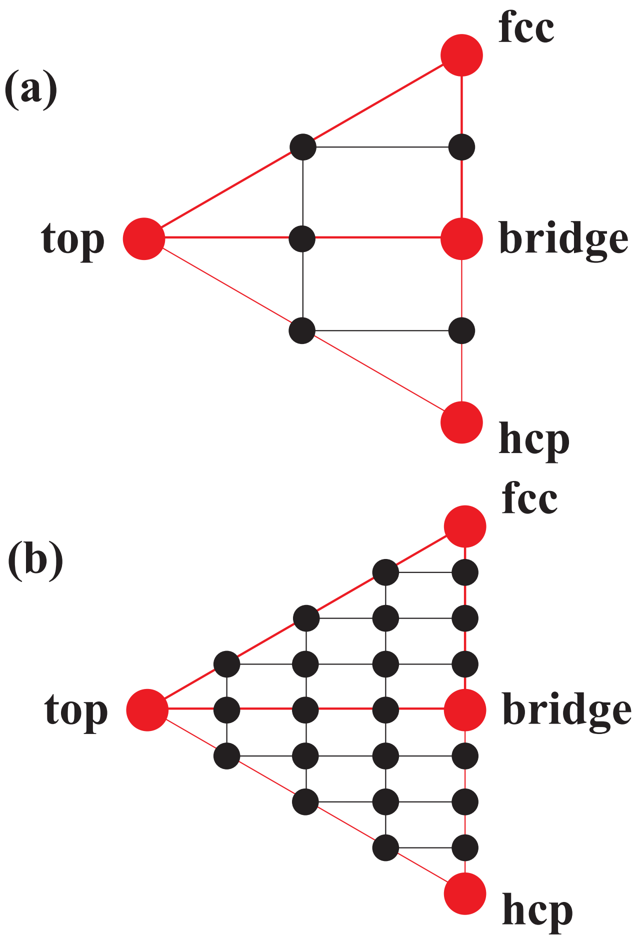

Supplementary Figure 1. Impact sites used in site-avearaging. (a) The schematic of the distribution of 9 sites (4 red circles denote the bridge, hcp, fcc, and top sites, and 5 black circles denote the midpoints of the adjacent sites). (b) The schematic of the distribution of 25 sites (16 additional sites correspond to the midpoints of the adjacent sites of the 9 sites shown above).

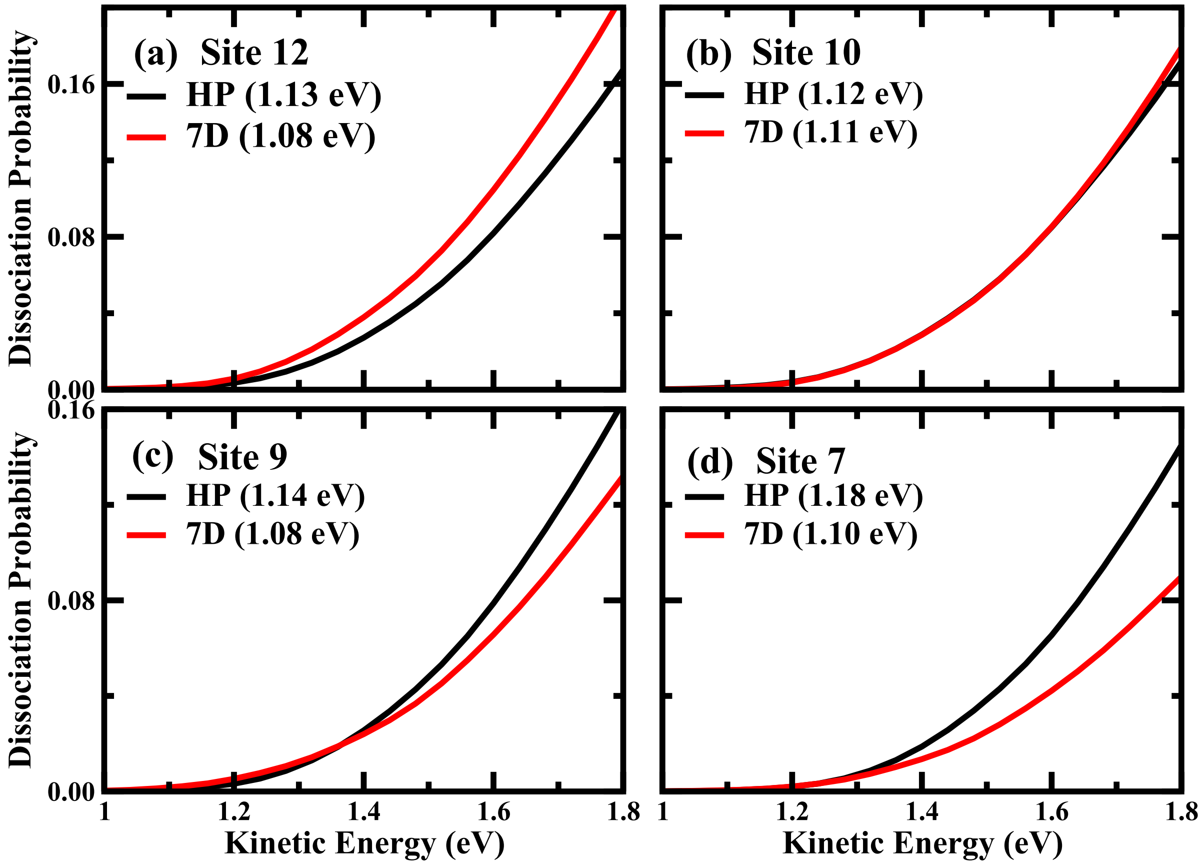

Supplementary Figure 2. Results with HP approximation and with the exact potential. (a-d) Comparisons of Site-specific dissociation probabilities obtained by the harmonic potential (HP) approximation and those calculated by 7D quantum dynamics calculations at the sites 12, 10, 9 and 7, respectively, with H_2O initially in the ground rovibrational state. The numbering of the sites are indicated in Fig. 4(a) in the paper. The barrier heights obtained by the HP approximation and the exact fitted PES are also indicated in the parentheses.

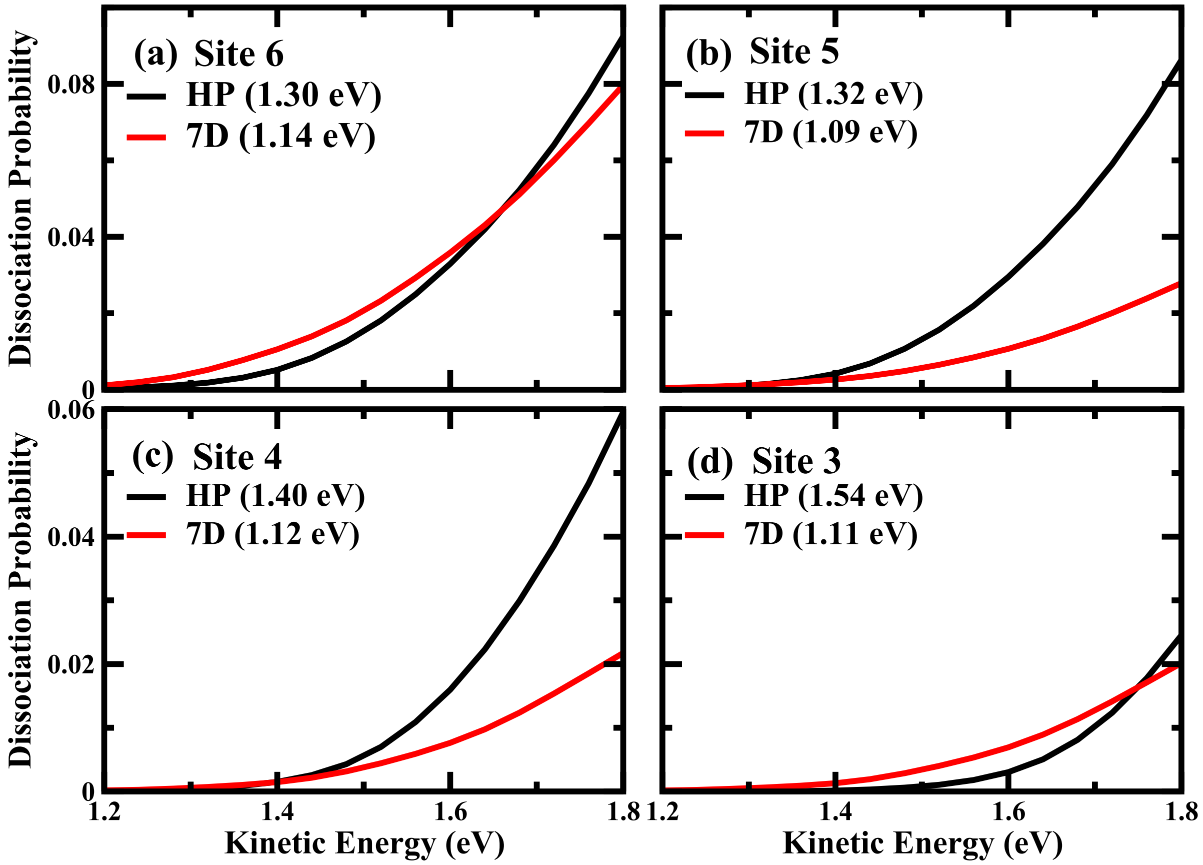

Supplementary Figure 3. Results with HP approximation and with the exact potential. (a-d) Same as Supplementary Figure 2, except for sites 6, 5, 4 and 3, respectively.

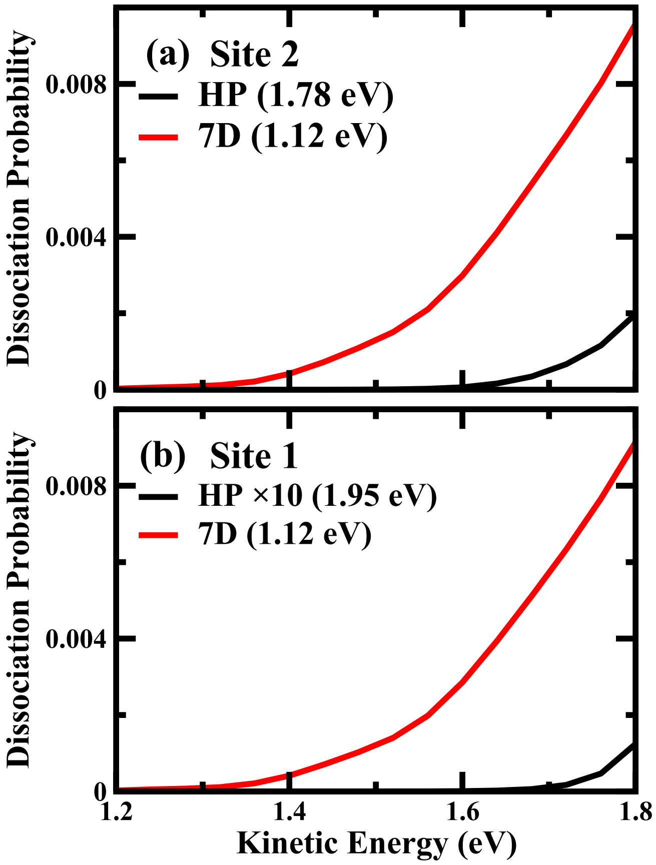

Supplementary Figure 4. Results with HP approximation and with the exact potential. (a-b) Same as Supplementary Figure 2, except for sites 2 and 1, respectively.

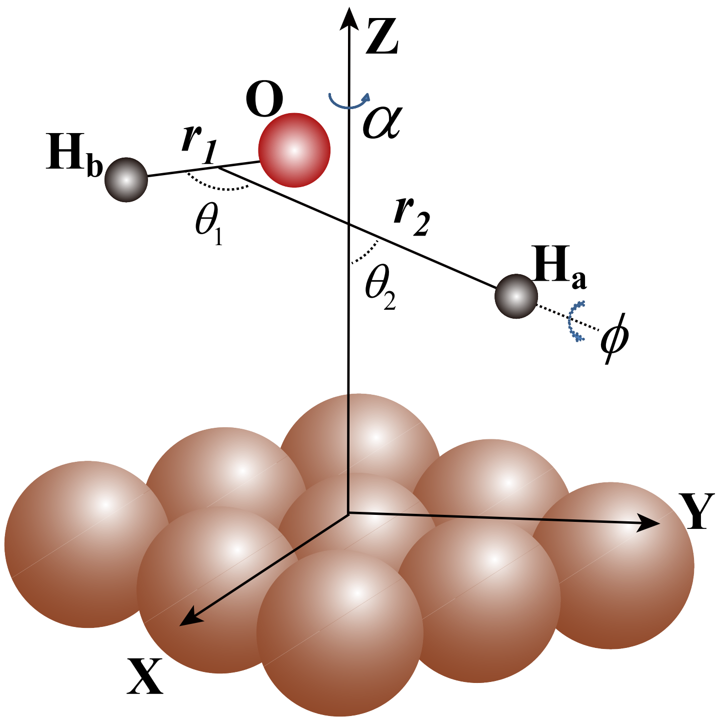

Supplementary Figure 5. Molecular coordinates. Definition of the molecular coordinates used in studying the dissociative chemisorption of H_2O on Cu(111).

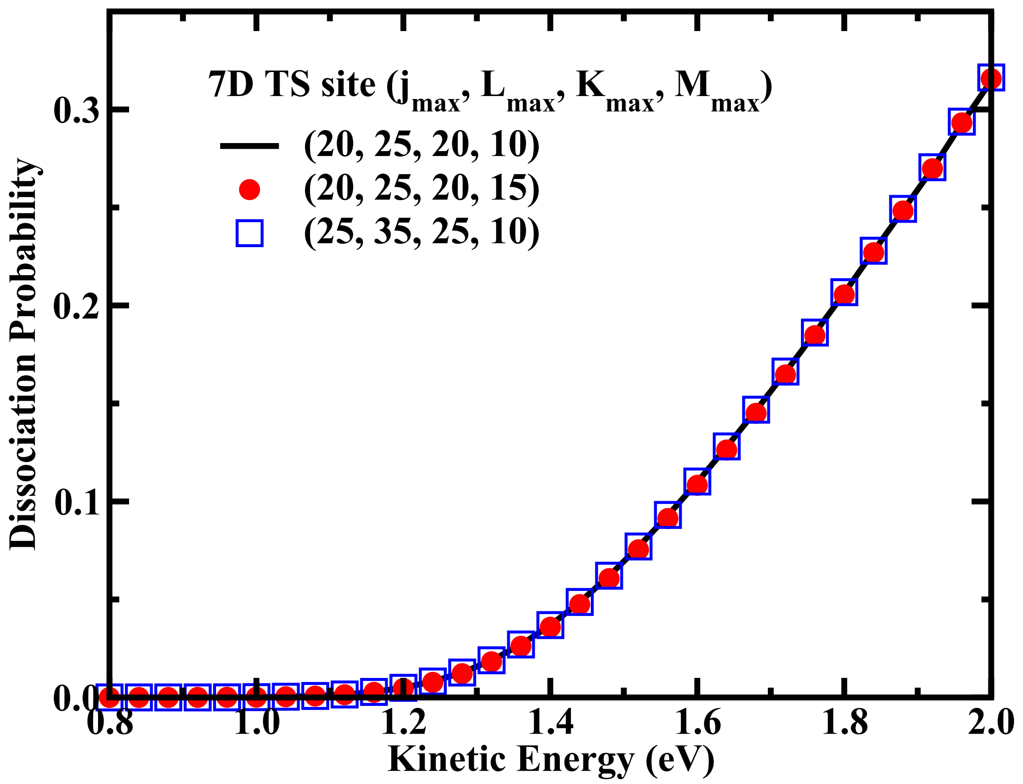

Supplementary Figure 6. Convergence of 7D dissociation probability. The convergence of 7D dissociation probability at the TS site with respect to the rotational basis.

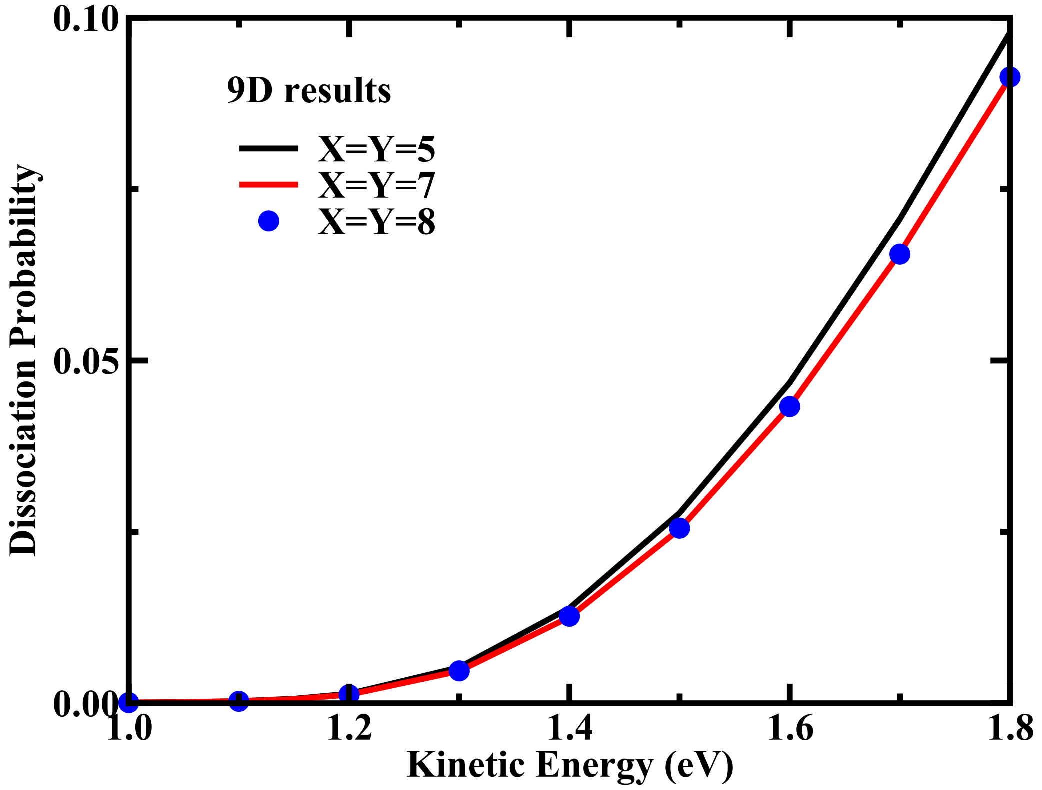

Supplementary Figure 7. Convergence of 9D dissociation probability. The convergence of 9D dissociation probability with respect to X and Y basis sets.

II. SUPPLEMENTARY DISCUSSION

A. Site-averaging approximation with exact potential (SAEP)

Early in 1997, Dai and Light found that the dissociation probabilities from the 4D fixed-site quantum dynamics calculations are quite different from those from 6D calculations in the H_2+Cu(111) system[1]. In addition, they averaged the 4D fixed-site dissociation probabilities for H_2 initially in the ground rovibrational state ($v = 0$, $j = 0$) over three impact sites (bridge, center, and top) to obtain the site-averaging dissociation probability, which is similar in shape with the 6D dissociation probability, while the latter is shifted to higher energy by about 0.05 eV. They assumed this energy shift was caused by the zero point energy (ZPE) differences between the 4D and 6D calculations at the transition states, as the 6D calculation incudes the ZPE for the two lateral coordinates x and y. However, the shifted 4D site-averaging dissociation probability is substantially smaller than the 6D dissociation probability at high kinetic energy. As a result, the explanation that ZPE differences account for the 4D to 6D shift in energy is not convincing.

Recently, we carried out the 4D fixed-site and 6D quantum dynamics calculation for the HCl+Au(111) and DCl+Au(111) reactions[2, 3], based on the LFZ PES developed by neural network fitting to DFT energy points[4]. A new finding for the site-averaging approximation with exact potential (SAEP) was presented for both reactions, i.e., the 6D dissociation probability can essentially be reproduced without ZPE corrections by averaging the 4D site-specific dissociation probabilities over impact sites, as long as sufficient impact points are used in the averaging. The validity of SAEP also holds in the H_2/Cu(111) reaction[5].

In this work, we employed our SAEP to approximate the full-dimensional reactivity as has been done in diatomic molecule-surface reactions[2, 3, 5]. Exploiting the C_{3v} symmetry (the p3m1 plane group symmetry) of the Cu(111) surface[6], it is sufficient to map the PES only for the molecular configurations inside the irreducible triangle of the surface unit cell which is spanned by the top, hcp, and fcc sites. Supplementary Figure 1 (a) shows that five midpoints of the top-hcp, top-fcc, top-bridge, bridge-hcp, and bridge-fcc lines, and the original 4 fixed sites (bridge, hcp, fcc, and top) constitute the present 9 sites. Similarly, the 25 sites consist of 16 midpoints of the two adjacent sites of the 9 sites mentioned above and the 9 fixed sites, as shown in Supplementary Figure 1 (b). We obtained the site-averaging

dissociation probability over 9 impact sites from the 7D fixed-site dynamical calculations with appropriate relative weights (3 for the bridge site, 1 for the hcp site, 1 for the fcc site, 1 for the top site, 3 for the four sites on the boundary of the triangle, and 6 for the site inside the triangle). Similarly, the 25 site-averaging dissociation probability was obtained using the similar relative weights.

B. Site-averaging approximation with harmonic potential (SAHP)

Jackson and co-workers have extensively studied the site and lattice effects for dissociative chemisorption dynamics, and their treatments within the sudden approximation have successfully reproduced the experimentally observed surface temperature dependence of dissociative sticking coefficients[7–9]. With that approximation, the potentials at various impact sites are approximated by a harmonic expansion around one specific site. We termed it the site-averaging approximation with harmonic potential (SAHP). Guo and coworkers also employed the SAHP to consider the effects of impact sites[10] for their 6D quantum dynamical results. The assumption made in the SAHP indicates that probabilities at different impact sites have the same energy dependence, but varying with the barrier height.

Briefly, the barrier corrugation is approximately like a harmonic oscillator and described as the following equation:

$$\Delta V(X,Y) = M\{\omega_x^2(X - X_{TS})^2 + \omega_y^2(Y - Y_{TS})^2\}/2, \quad (1)$$

where X_{TS} and Y_{TS} are the lateral coordinates of the transition state, M is the mass for H_2O and $\Delta V(X,Y)$ is the difference between the barriers at (X, Y) site and TS site. The ω_x and ω_y are the vibrational frequencies for lateral motion at the transition state and equals 141.65 and 184 cm^{-1} in this title system, respectively. Then the barrier height $V(X,Y)$ using the sudden approximation can be obtained:

$$V(X,Y) = V_{TS} - \Delta V(X,Y), \quad (2)$$

Where V_{TS} is the real static barrier height in the PES at the TS site and equals 1.08 eV for H_2O/Cu(111) system,

Following the sudden approximation, the dissociation probabilities at a specific (X, Y) site is approximated by shifting the $\Delta V(X,Y)$ probability:

$$P(E;X,Y) \approx P_0\{(E - \Delta V(X,Y)); X,Y\}, \quad (3)$$

where P_0 is the reaction probability at the TS site (In this work, P_0 is the 7D dissociation probability calculated using the TDWP method at the TS site), P is the probability at a specific site (X, Y) with the barrier shift $\Delta V(X, Y)$. Finally, the dissociation probability are obtained by averaging over the results for many (X,Y) sites which cover the surface unit cell, sampling all possible impact sites on the surface (Here, over 10000 impact sites are included in the averaging). The consequence of the site averaging is the shift of the dissociation probability curve along the energy axis.

C. Additional results

The comparisons made for the site-specific dissociation probabilities obtained by the harmonic potential (HP) approximation and by the 7D quantum dynamics calculations on the exact fitted PES at the rest of 10 impact sites are illustrated in Supplementary Figures 2-4, in the kinetic energy region of [0.8, 1.8 eV]. All the sites are labeled and presented in Fig. 4(a). The comparison at the top site is not illustrated because the dissociation probability obtained by the HP approximation in the energy region considered are completely zero, owing to the large barrier height (2.52 eV) from the HP approximation.

III. SUPPLEMENTARY METHODS

A. 9D potential energy surface (PES)

The full-dimensional potential energy surface (PES) was constructed by using the neural network fitting to a total of 81102 DFT energy points[11]. This fit results in an overall very small root mean square error (RMSE) of only 9.0 meV, but is signicantly smaller (6.0 meV) for energy points below 2.0 eV relative to the H_2O + Cu(111) asymptote, representing the unprecedented fitting accuracy for PESs of polyatomic-surface reactions. The resulting PES is accurate and smooth, based on the small fitting errors and good agreement between the fitted PES and the direct DFT calculations. All the planewave DFT calculations were carried out with the Vienna *ab initio* simulation package (VASP)[12, 13]. The interaction between ionic cores and electrons was described by fully nonlocal optimized

projector augmented-wave (PAW) potentials[14], and the Kohn-Sham valence electronic states were expanded in a planewave basis set[15]. The electron exchange correlation effects were treated within the generalized gradient approximation (GGA)[16], using the Perdew-Wang (PW91) functional[17]. The Cu(111) substrates consist of four layers with a 2×2 surface unit cell(1/4-ML coverage). A vacuum region between two repeated cells is 12 Å, the Monkhorst-Pack k-points grid mesh is 5×5×1[18] and the planewave expansion is truncated at the kinetic energy of 400 eV. The optimized lattice constants for bulk Cu is 3.636 Å in this work, which agrees well with the experimental value (3.615 Å)[19]. The PES is very well converged to the fitting process, examined by TDWP calculations. Readers can refer to our recent work for the details of the PES[11].

B. 9D Time-dependent wave packet (TDWP) approach

A total of 9 degrees of freedom (9D) should be considered for the fully coupled quantum dynamics calculations of the dissociative chemisorption of H_2O on a corrugated, rigid metal surface. The 9D Hamiltonian for the title reaction is expressed in terms of molecule coordinates (x, y, Z, r_1, r_2, θ_1, θ_2 , ϕ, α) (shown in Supplementary Figure 5) as,

$$\begin{aligned} \hat{H} = & -\frac{1}{2M}\left(\frac{1}{sin^2\Omega}\frac{\partial^2}{\partial x^2} - \frac{2cos\Omega}{sin^2\Omega}\frac{\partial^2}{\partial x\partial y} + \frac{1}{sin^2\Omega}\frac{\partial^2}{\partial y^2}\right) \\ & -\frac{1}{2M}\frac{\partial^2}{\partial Z^2} - \frac{1}{2\mu_1}\frac{\partial^2}{\partial r_1^2} - \frac{1}{2\mu_2}\frac{\partial^2}{\partial r_2^2} + \frac{j^2}{2\mu_1 r_1^2} + \frac{L^2}{2\mu_2 r_2^2} \\ & +V(x, y, Z, r_1, r_2, \theta_1, \theta_2, \phi, \alpha) \end{aligned} \tag{4}$$

where M is the mass of H_2O, μ_1 is the reduced mass of diatom OH_b , μ_2 is the reduced mass of diatom OH_b and atom H_a, r_1 is the bond length of non-dissociative OH_b bond, r_2 is the distance from the COM of diatom OH_b and atom H_a, Z is the vertical distance between the COM of H_2O and the surface, j is the rotational angular momentum operator for diatom OH_b and L is the orbital momentum operator of H_a with respect to diatom OH_b. The last term $V(x, y, Z, r_1, r_2, \theta_1, \theta_2, \phi, \alpha)$ is the interaction potential energy. Here the skewing angle Ω equals 120° for the Cu(111) surface.

The time-dependent wave function is expanded in terms of translational basis of Z, Fourier basis functions of X and Y, $\phi_{v_1}(r_1)$, $\phi_{v_2}(r_2)$and angular momentum eigenfunctions

$Y_{jL}^{JM}(\theta_1, \theta_2, \phi, \alpha)$ as

$$\Psi(x, y, Z, r_1, r_2, \theta_1, \theta_2, \phi, \alpha, t) = \sum_{n,v,j,L,M} F_{nvjLM}(t) u_n^v(Z) \phi_{v_1}(r_1) \phi_{v_2}(r_2) Y_{jL}^{JM}(\theta_1, \theta_2, \phi, \alpha) e^{ik_x x} e^{ik_y y} \quad (5)$$

where u_n^v is the translational basis function for Z which is dependent on v as given in Ref[20].

The total angular momentum basis $Y_{jL}^{JM}(\theta_1, \theta_2, \phi, \alpha)$ in Eq. (2) are defined as

$$Y_{jL}^{JM}(\theta_1, \theta_2, \phi, \alpha) = \sum_K D_{MK}^{J*}(\phi, \theta_2, \alpha) \sqrt{\frac{2L+1}{4\pi}} \times \langle jKL0|JK\rangle y_{jK}(\theta_1, 0) \quad (6)$$

where $D_{MK}^{J*}(\phi, \theta_2, \alpha)$ is the Wigner rotation matrix and y_{jK} are spherical harmonics. K is the projection of J(rotational angular momenta of H_2O) onto the H_a-OH_b axis. M is the projection of J on the surface normal.

The wave function is propagated using the split-operator method[21] and the time-dependent wave function is absorbed at the edges of the grid to avoid boundary reflections[22].

The initial state-selected total dissociation probability is obtained by projecting out the energy dependent reactive flux. If ψ_{iE}^+ denotes the time-independent (TI) full scattering wave function, where i and E are, respectively, initial state and energy labels, the total reaction probability from an initial state i can be obtained by the formula

$$P_i^R =< \psi_{iE}^+ \mid \hat{F} \mid \psi_{iE}^+ >, \quad (7)$$

In the above equation, $\hat{F}$ is the flux operator, defined as

$$\hat{F} = \frac{1}{2}[\delta(\hat{s} - s_0)\hat{v}_s + \hat{v}_s \delta(\hat{s} - s_0)] \quad (8)$$

where s is the coordinate perpendicular to a surface located at s_0 for flux evaluation, and $\hat{v}_s$ the velocity operator corresponding to the coordinate s. The full TI scattering wave function is normalized as $< \psi_{iE}^+ \mid \psi_{iE'}^+ >= 2\pi\delta(E - E')$. Using the expression in (5), Eq.(4) can be simplified to yield

$$P_i(E) = \frac{\hbar}{\mu_2} \text{Im}(\left\langle \psi_{iE}^+ | \psi_{iE}^{+'} \right\rangle)|_{s=s_0}, \quad (9)$$

where

$$\left|\psi_{iE}^+\right\rangle = \frac{1}{a_i(E)} \int_0^\infty e^{i(E-H)t/\hbar} \left|\Psi_i(0)\right\rangle dt, \quad (10)$$

with $a_i(E) = \langle \phi_{iE} | \Psi_i(0) \rangle$ being the overlap between the initial wave packet $\Psi_i(0)$ and the energy-normalized asymptotic scattering function ϕ_{iE}.

The numerical parameters used in the 9D quantum dynamics calculations are as follows: We used 300 and 30 sin-type DVR[23, 24] points to describe Z and r_2 coordinates, ranging from 2.0 to 14.5 bohrs and 1.2 to 5.5 bohrs, respectively. The non-reactive r_1 coordinate was described with 3 PODVR points (5 points for excited states), between 1.2-3.5 bohrs. The j_{max}, L_{max}, K_{max} and M_{max} equals 20, 25, 20 and 10 (L_{max} increases to 30 for excited states), respectively. The imaginary absorbing potentials are placed in the range of Z between 12.5 and 14.5 bohrs and r_2 between 4.0 and 5.5 bohrs, respectively, and the dissociation flux is calculated on the dividing surface of r_2=3.5 bohr. The time step for the propagation is 10 a.u. and we propagate the wave packets for 15000 a.u. of time to converge the dissociation probabilities at low energies. For the lateral x and y coordinates, the two dimensional unit cell formed by x and y is covered by a 7×7 evenly spaced grid. The numerical parameters of the 7D calculations are the same with the those of the 9D calculations except for x and y. Due to the non-reactive of one OH bond in current theory, the computed dissociation probabilities were multiplied with a factor of 2 which correspond to the results for two reactive bonds. The convergence of 7D dissociation probability at TS site with respect to the rotational basis, and of 9D probability with respect to X and Y, are shown in Supplementary Figure 6 and Supplementary Figure 7, respectively. The results converge quite well, indicating the current 9D calculations represent a true benchmark.

IV. SUPPLEMENTARY REFERENCES

---

[1] Dai, J. & Light, J. C. Six dimensional quantum dynamics study for dissociative adsorption of H_2 on Cu(111) surface. *J. Chem. Phys.* **107**, 1676–1679 (1997).

[2] Liu, T., Fu, B. & Zhang, D. H. Six-dimensional quantum dynamics study for the dissociative adsorption of DCl on Au(111) surface. *J. Chem. Phys.* **140**, 144701 (2014).

[3] Liu, T., Fu, B. & Zhang, D. H. Six-dimensional quantum dynamics study for the dissociative adsorption of HCl on Au(111) surface. *J. Chem. Phys.* **139**, 184705 (2013).

[4] Liu, T., Fu, B. & Zhang, D. H. Six-dimensional potential energy surface of the dissociative chemisorption of HCl on Au(111) using neural networks. *Sci. China Chem.* **57**, 147–155 (2013).

[5] Liu, T., Fu, B. & Zhang, D. H. Validity of the site-averaging approximation for modeling the dissociative chemisorption of H_2 on Cu(111) surface: A quantum dynamics study on two potential energy surfaces. *J. Chem. Phys.* **141**, 194302 (2014).

[6] Frankcombe, T. J., Collins, M. A. & Zhang, D. H. Modified Shepard interpolation of gas-surface potential energy surfaces with strict plane group symmetry and translational periodicity. *J. Chem. Phys.* **137**, 144701 (2012).

[7] Nave, S. & Jackson, B. Methane dissociation on Ni(111) and Pt(111): energetic and dynamical studies. *J. Chem. Phys.* **130**, 054701 (2009).

[8] Jackson, B. & Nave, S. The dissociative chemisorption of methane on Ni(111): the effects of molecular vibration and lattice motion. *J. Chem. Phys.* **138**, 174705 (2013).

[9] Farjamnia, A. & Jackson, B. The dissociative chemisorption of water on Ni(111): Mode- and bond-selective chemistry on metal surfaces. *J. Chem. Phys.* **142**, 234705 (2015).

[10] Hundt, P. M., Jiang, B., van Reijzen, M. E., Guo, H. & Beck, R. D. Vibrationally promoted dissociation of water on Ni(111). *Science* **344**, 504–507 (2014).

[11] Liu, T., Zhang, Z., Fu, B., Yang, X. & Zhang, D. H. A seven-dimensional quantum dynamics study of the dissociative chemisorption of H_2O on Cu(111): Effects of azimuthal angles and azimuthal angle-averaging. *Chem. Sci.* **7**, 1840–1845 (2016).

[12] Kresse, G. & Furthmuller, J. Efficiency of ab-initio total energy calculations for metals and semiconductors using a plane-wave basis set. *Comp. Mater. Sci.* **6**, 15–50 (1996).

[13] Kresse, G. & Furthmller, J. Efficient iterative schemes for ab initio total-energy calculations using a plane-wave basis set. *Phys. Rev. B* **54**, 11169–11186 (1996).

[14] Blöchl, P. E. Projector augmented-wave method. *Phys. Rev. B* **50**, 17953–17979 (1994).

[15] Kresse, G. & Joubert, D. From ultrasoft pseudopotentials to the projector augmented-wave method. *Phys. Rev. B* **59**, 1758–1775 (1999).

[16] Perdew, J. P., Burke, K. & Ernzerhof, M. Generalized Gradient Approximation Made Simple. *Phys. Rev. Lett.* **77**, 3865–3868 (1996).

[17] Perdew, J. P., Jackson, K. A., Pederson, M. R., Singh, D. J. & Fiolhais, C. Atoms, molecules, solids, and surfaces: Applications of the generalized gradient approximation for exchange and correlation. *Phys. Rev. B* **46**, 6671–6687 (1992).

[18] Monkhorst, H. J. & Pack, J. D. Special points for Brillouin-zone integrations. *Phys. Rev. B* **13**, 5188–5192 (1976).

[19] Haynes., W. M. (ed.). *CRC Handbook of Chemistry and Physics* (CRC Press, New York, 2013).

[20] Fu, B. & Zhang, D. H. A full-dimensional quantum dynamics study of the mode specificity in the H + HOD abstraction reaction. *J. Chem. Phys.* **142**, 064314 (2015).

[21] Fleck, J. A., J., Morris, J. R. & Feit, M. D. Time-dependent propagation of high energy laser beams through the atmosphere. *Appl. Phys.* **10**, 129–160 (1976).

[22] Neuhasuer, D. & Baer, M. The time-dependent Schrödinger equation: Application of absorbing boundary conditions. *J. Chem. Phys.* **90**, 4351–4355 (1989).

[23] Colbert, D. T. & Miller, W. H. A novel discrete variable representation for quantum mechanical reactive scattering via the S-matrix Kohn method. *J. Chem. Phys.* **96**, 1982–1991 (1992).

[24] Bacic, Z. & Light, J. C. Theoretical Methods for Rovibrational States of Floppy Molecules. *Annu. Rev. Phys. Chem.* **40**, 469–498 (1989).
